# The Role of Gut Microbiota in Fish Viral Infection: Mechanisms and Microbiota‐Targeted Interventions

**DOI:** 10.1155/anu/9336162

**Published:** 2026-02-27

**Authors:** Qiong Zhao, Jianzhong Shao, Ye Chen, Hangjun Zhang

**Affiliations:** ^1^ College of Life and Environmental Sciences, Hangzhou Normal University, Hangzhou, 311121, Zhejiang, China, hznu.edu.cn; ^2^ College of Life Sciences, Zhejiang University, Hangzhou, 310058, Zhejiang, China, zju.edu.cn

**Keywords:** fish viral disease, gut microbiota, immune regulation, pathological mechanism

## Abstract

Viral diseases represent one of the major threats to the global aquaculture industry. In recent years, the relationship between gut microbiota and viral infections in fish has garnered increasing attention. The gut microbiota contributes critically to fish health and is involved in antiviral defense through immune regulation, secretion of microbial metabolites, as well as enhancement of barrier function. The gut microbiota, host immunity, and viral infection form a complex and dynamic interaction network. A substantial body of 16S rRNA and metabolomics correlation studies has indicated that viral infections can alter the gut microbiota in fish, while changes in the gut microbiota can, in turn, influence viral infection. In this review, we summarize the regulatory effects of gut microbiota on fish viral infections, explore the interactions between the gut microbiota, immune system, and viral pathogenesis, and discuss future research directions and potential application prospects. By outlining the three‐dimensional interaction network of “microbiota‐immune‐virus” in fish, this review not only lays a theoretical foundation for developing targeted microecological strategies for green disease control but also provides an evolutionary perspective for understanding host–microbe coevolution in vertebrates.

## 1. Introduction

Fish farming is a vital component of the global aquaculture industry and has expanded rapidly in recent years, making significant contributions to food security and economic development. Global fish farming production continues to grow, particularly in Asia, with countries such as China, India, and Vietnam being the main producers [1]. According to the Food and Agriculture Organization (FAO) of the United Nations, the global aquaculture production was estimated at ~120 million tons in 2023, accounting for nearly 50% of the total global fisheries production. With the development of the aquaculture industry, fish viral diseases in high‐density farming environments have become one of the biggest challenges faced by the industry, severely limiting its growth and causing significant economic losses. Traditional antiviral treatments often rely on chemical drugs or vaccines, but these methods tend to have drawbacks such as high costs and environmental pollution. Recent studies have shown that the fish gut microbiota, often described as a functional “second genome," plays a crucial role in antiviral defense by regulating immune homeostasis, enhancing barrier functions, and directly inhibiting pathogens [2–4]. The composition and diversity of the gut microbiota not only influence fish nutrition and immune function but may also affect the occurrence and development of fish viral diseases by regulating immune responses or directly interacting with viruses [5–7]. This review integrates cutting‐edge research to analyze the interaction network between the gut microbiota and viral diseases, and discusses its potential applications in aquaculture health management. We will systematically summarize: (1) the impact of viral infections on the gut microbiota in fish; (2) how the gut microbiota influences fish susceptibility to viruses; (3) the strength of evidence and technical challenges regarding the application of probiotics, prebiotics, and microbial metabolite interventions in viral disease prevention and control.

## 2. Gut Microbiota in Fish

### 2.1. The Composition and Function of Gut Microbiota

The gut microbiota in fish refers to the microbial community inhabiting the intestinal tract, comprising bacteria, fungi, viruses, protists, and other microorganisms [8]. In this review, the term “gut microbiota” primarily refers to the bacterial communities, as they constitute the most extensively studied component to date. The diversity and composition of the gut microbiota are influenced not only by the habitat of the fish but also by factors such as their diet, taxonomic classification, and host‐specific selective pressures [9, 10]. The salinity and trophic levels of the fish’s habitat significantly impact the diversity and composition of the gut microbiota [9]. Microbial communities in the environment can serve as a source of inoculum for the fish gut microbiota, but the selective pressure exerted by the host plays a more critical role in shaping the gut microbiota [11]. Under different environmental conditions, the diversity and structure of the gut microbiota in fish can change significantly, which may be related to the nutrients and microbial communities present in the environment [12, 13].

The gut microbiota of fish shares similarities with that of other animals but also exhibits unique characteristics, suggesting that fish may have developed specialized symbiotic relationships during evolution [10]. At the functional level, the fish gut microbiota plays a crucial role in the host’s nutritional metabolism, immune regulation, and disease defense. The gut microbiota can influence the energy balance of fish by regulating lipid metabolism [14], and the functional diversity and redundancy of the gut microbiota vary among different fish species, providing stability to the ecosystem [15]. The gut microbiota interacts with the host’s immune system to help the host defend against pathogen invasion [16]. Thus, gut microbiota can influence the physiological functions, development, immunity, and pathogen resistance of fish.

### 2.2. Gut Microbiota in Fish Immunity

The gut microbiota is closely associated with the fish immune system. It plays an important role in the development and maturation of the immune system in fish. Studies have shown that the diversity and stability of the gut microbiota are closely related to the development of the fish immune system. In zebrafish, as the fish develops, the interactions and stability of the gut microbiota significantly increase, which may be attributed to the development of the fish’s immune system, the increased space available in the gut for microbial colonization, and the enhanced stability of nutrients [17]. Under sterile conditions, the development of gut‐associated lymphoid tissue (GALT) in zebrafish is affected, while a normal gut microbiota can promote the development of these tissues and enhance the fish’s immune defense capabilities [12]. Moreover, gut microbiota directly affects the immune response in fish by influencing intestinal barrier function, promoting the development and activation of immune cells, as well as regulating immune factors. Pathogenic bacteria like *Vibrio* sp. and *Aeromonas* sp. can activate immune responses by triggering the expression levels of proinflammatory cytokines in germ‐free and conventionally reared zebrafish [18]. However, some probiotics can enhance the protective immune response by regulating the composition of the intestinal flora. *Cetobacterium somerae* has been reported to improve liver and gut health by enhancing antiviral immunity [19]. And some probiotics containing high levels of α‐Gal can effectively inhibit *Mycobacterium marinum* infection, suggesting that probiotics exert their effects by modulating the gut microbiota and inducing protective immune responses [20]. An increasing body of evidence suggests that dysbiosis of the gut microbiota leads to the disruption of the intestinal barrier function, affecting immune function and contributing to the onset of diseases in fish [21, 22]. In largemouth bass (*Micropterus salmoides*), increased dietary viscosity significantly alters the structure and diversity of the gut microbiota, elevates the abundance of opportunistic pathogens, consequently disrupts microbiota homeostasis, compromises intestinal barrier function, and induces intestinal inflammation [22]. Deletion of Trmt5 in zebrafish results in epithelial disruption, gut microbiota disorder and immune system overactivation, which exhibits a spontaneous inflammatory bowel disease‐like phenotype [21]. Moreover, gut microbiota plays a crucial role in the fish’s anti‐pathogen immunity by influencing host immune tolerance and antiviral capabilities [3, 23]. Following SVCV infection, alterations were observed in the mucosal microbiota of common carp (*Cyprinus carpio*), characterized by a loss of dominant commensal microorganisms and prolific colonization by opportunistic bacteria. These changes suggest that secondary bacterial infections may occur in these mucosal tissues post‐viral infection, potentially implicating the mucosal microbiota in the antiviral defense mechanisms of fish [3]. MY Xue et al. [23] demonstrate that Aflatoxin B1 induced gut microbiota disorder to increase the infection of Cyprinid Herpesvirus 2 in Gibel Carp (*Carassius auratus gibelio*). Additionally, gut microbiota maintains intestinal homeostasis by modulating the fish immune system, which in turn detects and shapes the microbiota, thereby preserving intestinal health. Concurrently, commensal gut microorganisms contribute to intestinal health by regulating the host’s immune responses to suppress the overgrowth of pathogenic microbes [24]. The adaptive immune system serves as an ecological filter in zebrafish, promoting the persistence of specific microbial taxa, and consequently influencing the composition of the gut microbiota [25]. In summary, the role of the gut microbiota in fish immunity is multifaceted. It not only safeguards the host against pathogens through direct modulation of immune responses but also supports host physiological functions via its participation in metabolic processes.

## 3. Fish Viral Disease

### 3.1. Common Fish Viral Disease

Fish viral diseases are a group of serious diseases caused by viral infections, which severely affect aquaculture. They are one of the major constraints to the healthy development of the aquaculture industry. Fish viral diseases are a major infectious threat to aquaculture, characterized by high infectivity, rapid transmission, and high mortality rates. Outbreaks and epidemics of fish viral diseases pose one of the most significant threats to aquaculture, especially intensive farming, resulting in substantial economic losses. With the rapid development of aquaculture, the variety and spread of fish viral diseases are continuously expanding [26]. These viral diseases not only affect the health and yield of farmed fish but may also spread to wild fish populations through various channels, further intensifying the pressure on ecosystems. A number of viral diseases pose significant threats to aquaculture, such as grass carp hemorrhagic disease (GCHD), spring viremia of carp (SVC), infectious hematopoietic necrosis (IHN), carp edema virus disease (CEVD), and viral haemorrhagic septicaemia (VHS), among others. (Table 1). Grass carp reovirus (GCRV), a dsRNA virus, causes severe hemorrhagic disease in juvenile grass carp (*Ctenopharyngodon idella*), which is the largest production of fish in the world, affecting the cultivation industry affecting the cultivation industry [41]. SVC, caused by a rhabdovirus, SVC virus (SVCV), is an important disease affecting cyprinids, mainly common carp *Cyprinus carpio*, which is widespread in European carp culture and causes significant morbidity and mortality [28]. IHN is a severe rhabdoviral disease of farmed, hatchery and infrequently wild salmonids, which is caused by IHN virus (IHNV) belonging to the genus Novirhabdovirus of the Rhabdoviridae family. In the Pacific Northwest of North America, IHNV causes mass mortality in Chinook salmon (*Oncorhynchus tshawytscha*), kokanee (*O. nerka*), sockeye (anadromous *O. nerka*), rainbow trout (*O. mykiss*), and steelhead trout (anadromous *O. mykiss*). Juvenile fish infected with IHNV exhibit clinical symptoms such as lethargy, darkening of the skin, exophthalmos (bulging eyes), hemorrhages, and ascites. The pathogenesis of this virus includes degenerative necrosis of the kidneys and other hematopoietic tissues. Infectious hematopoietic necrosis disease can result in mortality rates of up to 100% in juvenile salmon under severe outbreak conditions [42, 43]. VHS Virus (VHSV) is a significant pathogen commonly found in fresh and saltwater fish worldwide such as rainbow trout, which can be transmitted through water and infected fish waste, with the virus having a longer survival time in the environment, especially during cold and low‐light winter months [44]. Another common fish viral disease is CEV‐induced carp edema disease. This disease not only leads to high mortality rates in fish but also causes severe metabolic disturbances, affecting the physiological balance of the fish [45]. Different types of viruses invade hosts and trigger immune responses through distinct mechanisms. The complexity and diversity of fish viral diseases present a severe challenge to both the aquaculture industry and ecosystems.

**Table 1:** Common fish viral disease.

Viral disease	Virus	Species of infected fish	Reference
Grass carp hemorrhagic disease (GCHD)	Grass carp reovirus (GCRV)	Grass carp and black carp	[27]
Spring viremia of carp (SVC)	Spring viremia of carp virus (SVCV)	Cyprinids	[28]
Herpesviral hematopoietic necrosis disease (HVHN)	Cyprinid herpesvirus 2 (CyHV‐2)	Goldfish and Crucian carp	[29]
Infectious Hematopoietic Necrosis (IHN)	Infectious hematopoietic necrosis virus (IHNV)	Salmonid species including Atlantic salmon and rainbow trout, and so on	[30]
Viral nervous necrosis (VNN)	Red grouper nervous necrosis virus (RGNNV)	Groupers and other marine fishes	[31]
Viral encephalopathy and retinopathy (VER)	Viral nervous necrosis virus (VNNV)	Marine and freshwater species	[32]
Infectious pancreatic necrosis (IPN)	Infectious pancreatic necrosis virus (IPNV)	Salmonid	[33]
Koi herpesvirus disease (KHVD)	Koi herpesvirus (KHV)	Carp *Cyprinus carpio*	[34]
Viral haemorrhagic septicaemia (VHS)	Viral haemorrhagic septicaemia virus (VHSV)	Marine fish	[35]
Carp edema virus disease (CEVD)	Carp edema virus (CEV)	Koi and common carp *Cyprinus carpio*	[36]
Iridoviridae disease	Iridoviridae	Bony fish	[37]
Salmonid Alphavirus disease (SAD)	Salmonid Alphavirus (SAV)	Atlantic salmon and rainbow trout	[38]
Infectious salmon anaemia (ISA)	Infectious salmon anaemia virus (ISAV)	Atlantic salmon	[39]
Infectious spleen and kidney necrosis (ISKN)	Infectious spleen and kidney necrosis virus (ISKNV)	Freshwater and marine fish species	[40]

### 3.2. The Pathological Mechanism of Fish Viruses

The pathological mechanisms of fish viruses are a complex field, involving various virus invasion, replication, host immune responses, and pathological changes. Different types of fish viruses induce diseases through multiple mechanisms, causing severe impacts on the aquaculture industry. Fish viruses invade host cells through specific mechanisms, typically relying on the binding of viral surface proteins to host cell surface receptors. Different types of fish viruses have distinct cell tropism, affecting different tissues and organs. For example, Koi Herpesvirus (KHV) initially invades the gill epithelial cells [46]; Cyprinid herpesvirus 2 (CyHV‐2) primarily attacks the hematopoietic tissues in the spleen and kidneys [29]; Tilapia Lake Virus (TiLV) can infect liver cells [47], and so on. Once a virus enters a host cell, it utilizes the host’s cellular machinery to replicate and synthesize new viral particles. The viral genome (typically RNA or DNA) is released into the nucleus or cytoplasm, where viral proteins and new viral RNA are synthesized through the host’s transcription and translation processes. DNA viruses (such as Cyprinid herpesvirus 3, CyHV‐3) typically replicate within the cell nucleus. RNA viruses (such as IHNV and VHSV) replicate in the cytoplasm. Among them, retroviruses (such as fish sarcoma viruses) require reverse transcription into DNA and integration into the host genome. New viral particles accumulate within the cell, ultimately causing rupture (lysis) of the host cell. This releases numerous viruses, enabling them to infect additional cells. Viral infections directly lead to the hijacking and depletion of immune cells. In TiLV infections, granulocytes are infected by the virus, enabling systemic dissemination. At the same time, lymphocytes undergo apoptosis, weakening the adaptive immune response [48]. Viral infections typically trigger the host’s innate immune response, including the recognition of viral nucleic acids or proteins by toll‐like receptors (TLRs) or RIG‐I‐like receptors (RLRs), which activate the production of interferons (IFNs) and other antiviral proteins [49]. Immune cells, such as macrophages and neutrophils, migrate to the site of infection in an attempt to clear the virus. In some viral infections, the immune response itself can lead to tissue damage, and an excessive cytokine storm may cause inflammation and necrosis, further exacerbating clinical symptoms. Additionally, fish viruses often evade host immune surveillance through various mechanisms, allowing the virus to persist in the body. Viruses weaken the host’s antiviral immune response by preventing the production of IFNs and other antiviral factors. For instance, GCRV suppresses Type I IFN production via the IL6‐STAT3‐HSP90 signaling axis, with activity regulated by temperature [50]. Viruses can evade immune detection by interfering with the host cell’s viral antigen presentation process [51]. Moreover, some viruses exhibit latent infection and reactivation. Herpesviruses (such as KHV and CyHV‐2) can remain latent in immune cells, and reactivation occurs under stress conditions (e.g., sudden changes in water temperature or transport), leading to intermittent outbreaks [46, 52]. Further, viral infections typically lead to necrosis of virus‐targeted cells, particularly in immune‐related tissues such as the liver, kidneys, spleen, and intestines [53–55].

### 3.3. Host Immunity and Fish Virus Infection

#### 3.3.1. Innate Immune Responses to Virus

**Figure 1:**
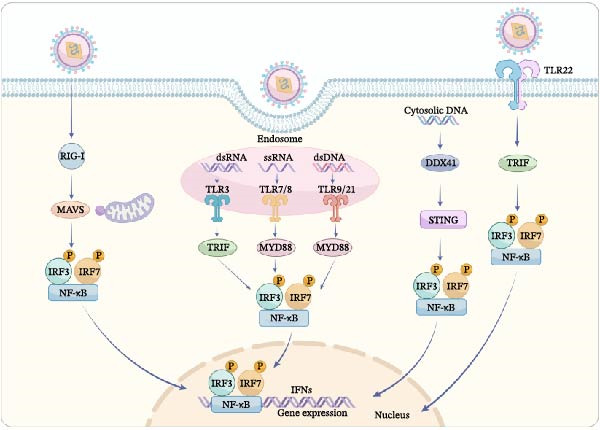
Virus‐mediated activation of innate immune signaling. The activation of TLR3, TLR7/8, TLR9/21, TLR22, Rig‐I, and DDX41 by dsRNA, ssRNA, dsDNA, and cytosolic DNA triggers signaling cascades. Adaptor proteins MyD88 and TRIF initiate pathways that activate IRF3, IRF7, and NF‐κB, leading to an antiviral response.

The immune system in fish has similarities to the mammalian immune system, which consists of innate immunity and adaptive immunity. Fish innate immune system launches the primary response to virus infection, characterized by a rapid and nonspecific response. Compared to higher vertebrates, although fish possess a fully functional adaptive immune system, its response is relatively slower, which makes innate immunity play a more crucial role in fish antiviral defense. Fish largely rely on the innate immune defense, which involves physical barriers, immune cells, and soluble immune factors [56]. The physical barriers, such as the skin, mucus and scales, can effectively prevent the initial invasion of pathogens. Immunocytes of the innate immune system in fish are composed of macrophages and neutrophils with morphological and functional similarities to mammals’ leukocytes, which exerts its antiviral function by phagocytosis and secretion of cytokines [57]. Other types of leukocytes such as nonspecific cytotoxic cells (NCCs), considered as an evolutionary precursor of mammalian natural killer cells, have been identified in fish, but their functionality has yet to be studied [58, 59]. Immune factors such as interferon regulatory factors (IRFs), cytokines, and so on, play significant roles in virus clearance [60]. Once viruses breach the physical barriers, the fish organism rapidly initiates immune responses at both cellular and molecular levels. Viruses produce nucleic acids during their replication, either during genomic replication or transcription. The viral nucleic acids are called pathogen‐associated molecular patterns (PAMPs) and recognized by pattern recognition receptors (PRRs), including TLRs, RLRs, cytoplasmic DNA sensors (CDSs), and class A scavenger receptors (SR‐As). Subsequently, this recognition activates downstream signaling transduction pathways, inducing the production of type I IFNs, inflammatory cytokines, and various antiviral proteins, thereby establishing an effective antiviral state [61, 62]. TLRs are the core molecules in the innate immune response of fish which activate the immune response by recognizing specific PRRs of exogenous viruses. While many TLRs exist in fish, their specific targets are often unknown. Based on experimental data, some TLRs, including TLR3, TLR7, TLR8, TLR9, TLR21, TLR22, and so on. have been proved to recognize virus nuclear acid (Figure 1). Among them, TLR3 mainly recognizes viral double‐stranded RNA; TLR7 and TLR8 recognize viral single‐stranded RNA; TLR9 and TLR21 are located in the endosome but recognize DNA; and TLR22 can recognize long‐chain double‐stranded RNA [63–66]. When the TLRs are activated, adaptor proteins, including MyD88 and TRIF, which mediate the inflammatory and type I IFN responses within the cell [65]. MyD88 binding to activated PRRs triggers NF‐ĸB activation and translocation, leading to pro‐inflammatory cytokine expression. Key proteins in the MyD88 pathway include IRAK‐4 and TRAF6, whose overexpression stimulates NF‐ĸB in zebrafish cells. Besides NF‐ĸB, MyD88 interacts with type I IFN‐pathway transcription factors IRF3 and IRF7. Most TLRs signal via MyD88, except TLR3 and TLR22, which use TRIF, found in many species of fish. TRIF activates NF‐ĸB and IRF3/IRF7 pathways, resembling its mammalian role in signaling and IFN production [67, 68]. As reported, TLR3 expression rises following poly I:C treatment or viral infection, enhancing pro‐inflammatory and IFN‐stimulated gene expression in various types of fish [56, 69, 70]. In Japanese flounder, TLR3 overexpression boosts pro‐inflammatory and IFN‐stimulated genes (ISGs) expression, while TLR3 knockdown reduces it, indicating TLR3’s role in antiviral activity against dsRNA, similar to mammals [57]. TLR22, unique to teleost fish and part of the TLR11 family, recognizes dsRNA and signals through TICAM to induce IFNs, functioning similarly to TLR3 [64]. Cytosolic nucleic acids are recognized by *retinoic acid-inducible gene I (RIG-I)*, mitochondrial antiviral signaling protein (MAVS), and laboratory of genetics and physiology 2 (LGP2), which detect viral dsRNA and activate MAVS for further signaling, collectively known as RLRs [71]. The interaction between RIG‐I and MAVS initiates a signaling cascade that activates IRF3, IRF7, and NF‐κB, resulting in the expression of type I IFNs and cytokines to inhibit viral replication. MAVS, upon recognizing PAMPs via RLRs, associates with TRAF3, STING, and TBK1, activating IRF3/IRF7 for type I IFN production. MAVS also activates TRAFs and an IKK complex to trigger NF‐*κ*B, promoting pro‐inflammatory cytokine production [72–74]. Moreover, cytosolic DNA is also detected by several intracellular receptors called CDSs (cytosolic DNA sensors). In fish, DDX41 is the sole CDS that activates STING and TBK1, key components in the antiviral response. STING, crucial for detecting cytosolic DNA, enhances type I IFN and ISG expression when overexpressed, but its absence weakens the innate antiviral defense, increasing vulnerability to viruses. STING can bind both self and foreign DNA, triggering antiviral and inflammatory gene activation [75]. Upon activation by cytosolic DNA, STING relocates from the endoplasmic reticulum to the Golgi, forming structures with TBK1 that may activate TBK1, leading to IRF3 phosphorylation and IFN production [76]. However, DDX41 remains the only CDS described thus far in fish. Whether the additional CDSs existing in fish need to be further studied. In summary, fish virus infection initiates the innate immune response.

#### 3.3.2. Adaptive Immune Responses to Virus

As the earliest vertebrates possessing an adaptive immune system, fish occupy a unique position in the evolution of immunity. Compared with mammals, the adaptive immune system of fish is more primitive, yet it retains the fundamental abilities of specific recognition and immune memory. The adaptive immunity of fish is mainly mediated by T lymphocytes and B lymphocytes, which combat viral infections through the production of specific antibodies and cellular immune responses. However, in contrast to higher vertebrates, the adaptive immune responses of fish are typically slower to initiate, weaker in intensity, and possess relatively limited immune memory. As a result, fish rely more heavily on the rapid responses of the innate immune system when facing viral invasion. Despite certain limitations in the adaptive immune system of fish, its fundamental immune components remain conserved with those of higher vertebrates. Fish possess major histocompatibility complex (MHC) molecules, diverse immunoglobulin types, and specialized immune cells, all of which enable them to mediate specific immune responses [77, 78]. B‐cell receptors (BCRs) and T‐cell receptors (TCRs) are key components of the adaptive immune system, recognizing foreign pathogens. This system enhances its response with repeated antigen exposure, forming the basis for vaccines by developing immunological memory. The adaptive response, which becomes effective days after infection, specifically targets foreign antigens and improves in speed and strength upon subsequent exposures [79]. B‐cells drive antibody responses, while T‐cells handle cell‐mediated responses; both crucial for antiviral defense and work together. Antigen‐presenting cells, like dendritic cells and macrophages, link the innate and adaptive immune systems by presenting processed antigens to T‐cells via MHC class 2 receptors, which in turn initiates the adaptive cell‐mediated immune response [60].

## 4. Gut Microbiota and Viral Disease in Fish

### 4.1. Dysbiosis of Gut Microbiota Affects the Viral Susceptibility of Fish

Mucosal surfaces in vertebrates constitute both physical and chemical barriers that separate the host from the external environment and harbor highly concentrated and complex microbial communities, which play vital roles not only in digestion and nutrition but also are critical for protecting the host against pathogens and environmental threats [80, 81]. As early vertebrates, fish exhibit a relationship between their intestinal microbiota and viral infections that has not yet been fully understood. Nevertheless, existing research demonstrates that the gut microbiota plays a pivotal role in the immune responses of fish, particularly during viral infections. The composition of the intestinal microbiota may directly influence the susceptibility of fish to viruses. Dysbiosis of the intestinal microbiota, characterized by reduced bacterial diversity and decreased presence of specific beneficial bacteria, has been associated with a weakened host immune response, potentially increasing the susceptibility of fish to viral infections and accelerating the spread of viruses [3, 82, 83]. Especially, in high‐density breeding environments with frequent antibiotic use, imbalanced intestinal flora has been linked to an increased risk of viral disease outbreaks. This imbalance in fish often correlates with enhanced viral replication, worsened disease progression, and immune evasion. Temperature affects host susceptibility and virus infectivity in aquaculture, with viruses like CyHV‐3, GCRV, ISKNV, NNV, and so on, being more infectious at high temperatures, while low temperatures increase susceptibility to viruses such as HIRRV, SVCV, and IPNV [84, 85]. Meanwhile, temperature also affected the composition of the intestinal microbiota in zebrafish, thereby influencing the infectivity and pathogenicity of SVCV [85]. Under controlled laboratory conditions in zebrafish, the temperature‐sensitive microbiota, such as *Parabacteroides distasonis*, which highly enriched in zebrafish exposed to high‐temperature environments, inhibits the assembly and release of SVCV. The use of antibiotics such as sulfamethoxazole (SMX) and clarithromycin (CLA) has also been proven to cause dysbiosis in the intestinal flora of zebrafish, thereby increasing their susceptibility to SVCV [86]. Moreover, dietary supplementation of probiotics improved the resistance of fish against virus infection [87, 88]. In summary, available evidence indicates that the balance of the intestinal microbiota is crucial for fish to resist virus infections. Whether it is the use of antibiotics or probiotics, it will affect the viral susceptibility of fish by altering the structure of the intestinal microbiota.

### 4.2. Gut Microbiota and Antiviral Immunity

**Figure 2:**
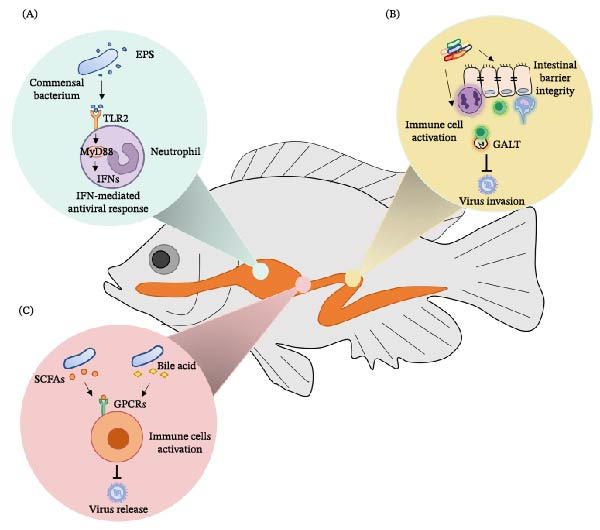
The mechanism of the gut microbiota regulating antiviral immunity in fish. (A) Commensal bacteria interacts with host pattern recognition receptors (PRRs) through microbial‐associated molecular patterns (MAMPs), continuously enhancing the IFN‐mediated antiviral response. (B) Gut microbiota interacts with the intestinal epithelial cells, immune cells, and gut‐associated lymphoid tissue (GALT), regulating the host’s immune response and playing an important role in defending against viruses. (C) Gut microbiota‐derived metabolites play a significant role in regulating antiviral immunity.

The gut microbiota plays a pivotal role in modulating antiviral immunity, acting as a crucial interface between the host and viral pathogens. The intricate relationship between gut microbiota and the immune system is underscored by the ability of specific microbial communities to influence both innate and adaptive immune responses. For instance, microorganisms in the intestines interact with host PRRs via microbial‐associated molecular patterns (MAMPs), continuously shaping the immune development and function of the host’s immune system, a process analogous to that observed in mammalian systems. In zebrafish, *Cetobacterium somerae* secretes extracellular polysaccharides (EPS), which activates the TLR2‐MyD88 signaling pathway, enhancing the response of neutrophils and the antiviral innate immunity mediated by type I IFNs, thereby inhibiting the infection of SVCV [89] (Figure 2A). Additionally, there is a complex interaction between the mucosal immune system and gut microbiota in fish. The gut serves as the first line of defense against many pathogens entering the host. Gut microbiota helps prevent the invasion of viruses and other pathogens by maintaining the integrity of the intestinal barrier. Furthermore, the microorganisms in the gut interact with the intestinal epithelial cells, immune cells, and GALT, regulating the host’s immune response and playing an important role in defending against pathogens (Figure 2B). The fish immune system can sense and modulate the gut microbiota to maintain intestinal homeostasis. At the same time, the gut symbiotic microorganisms can also regulate the immune response of the fish, inhibiting the overgrowth of pathogenic microorganisms and ensuring the health of the host’s gut [24, 90]. However, the gut microbiota’s impact on antiviral immunity is not limited to direct interactions with the immune system but also involves the production of metabolites. Gut microbiota‐derived metabolites have become key mediators in the host–microbe antiviral response, which not only act locally in the gut but also influence systemic immune defenses through the circulatory system, forming a novel class of “microbiome‐immune messengers”. The metabolites of gut microbiota, short‐chain fatty acids (SCFAs), regulate the host’s antiviral immune response through multiple mechanisms such as G protein‐coupled receptors (GPCRs), histone deacetylases (HDACs), and metabolic reprograming [91, 92]. In fish, research on metabolite‐mediated immunity has shown that specific gut bacteria can regulate bile acid metabolism through bile salt hydrolase (BSH), generating secondary bile acids with direct antiviral activity. Specifically, *Parabacteroides distasonis* produces the secondary bile acid deoxycholic acid (DCA), inhibiting the assembly and release of SVCV in zebrafish [85]. These metabolites play a significant role in regulating antiviral immunity and offer therapeutic potential for managing viral diseases and preparing for pandemics (Figure 2C). Overall, the gut microbiota is a critical regulator of antiviral immunity, influencing both local and systemic immune responses through a variety of mechanisms. The interplay between gut microbes, their metabolites, and the host immune system offers valuable insights into potential therapeutic strategies for enhancing antiviral defenses and managing viral infections. Understanding these complex interactions is essential for developing novel interventions that leverage the gut microbiota’s immunomodulatory capabilities.

### 4.3. Viral Infection Induces the Perturbation of Gut Microbiota

**Figure 3:**
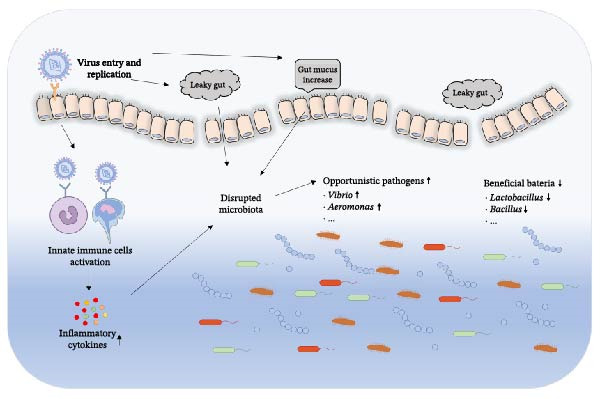
Viral infection induces the perturbation of gut microbiota through multiple pathways.

Besides the influence of gut microbiota on viruses, viral infection can also disrupt the homeostasis of gut microbiota [93]. For example, GCRV infection significantly reduced microbial diversity of grass carp with a lower Shannon index but with a much higher ecological process of homogenizing dispersal, confirming a dysbiosis of the gut microbiota after viral infection. Moreover, it decreased the abundance of key symbiotic bacteria (*Cetobacterium*), but increased the opportunistic pathogens (*Aeromonas*) [83]. Similarly, SVCV infection disturbed the composition of intestinal microbiota in carp with increased *Proteobacteria* and decreased *Fusobacteria* [3]. In gibel carp, the diversity and composition of the intestinal microbiota were strongly reduced following CyHV‐2 infection. Pathogenic bacteria like *Plesiomonas*, *Bacteroides*, and *Flavobacterium* were the most abundant species in sick fish, among which *Plesiomonas* was highly abundant in infected samples and could be used as a microbial biomarker for CyHV‐2 infection [94]. Viral replication and the associated immune responses can disrupt intestinal barrier function and disturb the homeostatic relationship between the host and its microbiota, leading to a reduction of beneficial bacteria (such as *Lactobacillus* and *Bacillus* spp.) and an expansion of opportunistic pathogens (such as *Vibrio* and *Aeromonas* spp.; Figure 3). This dysbiosis not only impairs intestinal nutrient metabolism and immune function but may also increase the risk of secondary bacterial infections, thereby exacerbating inflammatory responses and mortality in fish [83].

A virus does not directly target microbiota, but it alters the intestinal microenvironment through multiple mechanisms. First, following viral infection, the host immune system is activated and produces a large number of inflammatory cytokines, leading to an altered intestinal chemical microenvironment (e.g., pH and oxygen concentration), to which different bacterial taxa exhibit varying tolerances. Beneficial bacteria generally prefer a stable and mild environment, whereas many opportunistic pathogens are more tolerant of, or even able to exploit, inflammatory conditions. Consequently, the microbial community structure undergoes an ecological selection pressure, in which beneficial bacteria are outcompeted by opportunistic species [4, 95]. Some fish viruses, such as CyHV‐3, can directly infect and damage intestinal epithelial cells, which increase intestinal permeability and reduce the available surface area for bacterial adherence. Such physical damage directly changes the habitation which the bacteria rely for survival, resulting in the loss of ecological niches for the original microbiota [96]. The fish intestinal mucosa is among the main sites through which environmental microorganisms interact with the host, which not only constitutes the first line of defense against pathogenic microorganisms but also plays a crucial role in commensal colonization. IHNV successfully invaded the gut mucosa of trout, resulting in severe tissue damage, inflammation, and an increase in gut mucus, which induced severe dysbiosis of gut microbiota [4]. Moreover, viral infection alters the metabolism of fish, and the metabolic products can also affect the growth of the microbiota [97, 98]. In summary, the disturbance of the gut microbiota caused by viral infections in fish represents a typical “disruption‐replacement‐aggravation’ process: viral invasion triggers immune responses, inflammation and tissue damage, which alter the gut microenvironment and further induce microbial imbalance. The loss of intestinal barrier integrity and immune regulatory function facilitates viral replication and secondary infections, thereby worsening the disease in fish.

## 5. Gut Microbiota‐Related Treatment to Fish Viral Disease

In recent years, viral diseases in fish have occurred frequently, causing severe economic losses to the aquaculture industry [28]. Traditional prevention and control strategies, such as vaccination and drug treatment, have certain limitations. Research has shown that the intestinal microbiota plays an important role in fish health. The fish gut microbiota not only participates in digestion, absorption, and nutrient metabolism but also influences the progression of viral infections by regulating immune responses, producing antiviral metabolites, and competitively excluding pathogens [99, 100]. Therefore, intervention strategies targeting the gut microbiota, such as the application of probiotics, prebiotics, symbionts, and microbial metabolites, and so on, provide a new direction for the prevention and control of viral diseases in fish.

### 5.1. Probiotics

Probiotics are regarded as living microorganisms, which can enhance the host’s immune response and inhibit the growth of pathogenic bacteria by regulating the host’s gut microbiota, thereby improving the host’s resistance to pathogens [101]. A large number of studies have demonstrated that probiotics play an important role in the health and welfare of a variety of aquaculture species. The application of probiotics in the treatment of fish viral diseases has become a research hotspot in the field of aquaculture in recent years. Probiotics regulate the host’s immune system, enhancing the fish’s resistance to viral infections, thereby reducing the occurrence and spread of viral diseases. A study on TiLV presented that supplementing with probiotic *Bacillus* spp. could significantly reduce the mortality rate and viral load after infection, as well as promote the expression of immune‐related genes, indicating that probiotics can enhance the fish’s resistance to viral infections by strengthening the immune response [102]. Moreover, probiotic *Bacillus* has been reported to serve as an antiviral therapeutic agent against different viral infections in multiple fish [95, 103–105]. A comparative study shows that *Lactobacil*, individually or mixed with *Sporolac* acts, as immunostimulants that enhance the innate immune response and disease resistance in lymphocystis disease virus (LCDV) infected olive flounder [106]. Various species of *Lactobacillus*, including *Lactobacillus plantarum* and *Lactobacillus rhamnosus GCC-3*, improve the resistance of fish against virus infection [87, 107]. *Cetobacterium somerae* inhibits viral infection through TLR2‐type I IFN signaling axis in zebrafish [89]. Moreover, probiotics can also serve as vaccine carriers and exert antiviral effects during viral infections in fish. A recent study demonstrates that an oral probiotic vaccine loaded by *Lactobacillus casei* effectively increases defense against GCRV infection in grass carp [88]. Recombinant probiotics expressing virus proteins increase the resistance of relative virus infection, and this strain is a promising candidate oral vaccine for the prevention of virus infection in fish [95, 108, 109]. In conclusion, the application of probiotics in the treatment of fish viral diseases has a broad prospect. By regulating the host’s immune system, probiotics can enhance the fish’s resistance to viral infections, thereby reducing the incidence and mortality rates. However, in order to achieve its wide application in aquaculture, more research is still needed to optimize its application strategies.

### 5.2. Prebiotics

Prebiotics refer to substances that can promote the growth or activity of beneficial intestinal bacteria, which are typically carbohydrates that are difficult for the host to digest, such as dietary fiber, inulin, oligosaccharides of glucose (like β‐glucans), galactose, fructose, or mannose [110]. The application of prebiotics in the treatment of fish viral diseases has become an important research direction in the field of aquaculture in recent years. By selectively promoting the growth of probiotics, prebiotics indirectly improve the host’s immune system and enhance the resistance of fish to viral infections [6]. The use of prebiotics as immunostimulants in farmed fish feed has been reviewed elsewhere [111]; however, the effect of prebiotics on treating fish viral disease needs to be further examined. We summarized such studies in this review. Yin et al. [91] reported that supplementation of dietary crude lentinan played the antiviral effects on IHNV infection in rainbow trout through strengthening the intestinal immune barrier and modifying the aberrant changes of intestinal microbiota induced by IHNV, mainly represented by promoting the growths of *Cetoobacterium* and *Deefgea* and inhibiting *Mycobacterium* and *Nannocystis*. The plant‐derived alkaloid palmatine enhances the innate immune response in fish by activating key immune genes such as *IRF3* and *IFN7*, thereby significantly inhibiting the replication of *micropterus salmoides* rhabdovirus (MSRV). This immunoregulatory effect not only improves fish survival rates but also reduces transmission of the virus, highlighting its potential as a small‐molecule immunomodulator in aquaculture [112]. Poly‐β‐hydroxybutyrate (PHB) supplementation changed the microbial structure but not diversity, and significantly increased beneficial bacteria such as *Bacillus* sp, which upregulated transcriptional levels of immune‐related genes, decreased early CyHV‐2 replication in spleen and cumulative mortality of gibel carp [113]. Some functional feeds containing β‐glucans, vitamin C, vitamin E and zinc significantly upregulated immune‐related genes, including *IgM*, *IgT, IgD*, and *Mx*, which enhanced the resistance of rainbow trout to VHSV infections and reduce the impact of viral diseases in fish [114]. These findings reveal an undescribed effect of functional diets on fish Ig production and point to those functional feeds as an adequate diet to be incorporated in holistic programs aimed at mitigating the effect of viral diseases. Moreover, β‐glucan has been reported to change the composition of intestinal microbiota with upregulated abundance of probiotics and reduce virus infection in multiply fishes, including sevenband grouper, turbot, zebrafish, rainbow trout, *carassius auratus* gibelio, and so on [115–119]. According to those studies, prebiotics enhance fish immunity through multiple mechanisms, thereby playing an important role in the prevention and treatment of viral diseases. The application of prebiotics in aquaculture not only helps improve fish health but also provides new approaches for sustainable disease management. These findings lay the foundation for the future application of prebiotics in the treatment of viral diseases in fish and provide a scientific basis for the sustainable development of the aquaculture industry.

### 5.3. Microbial Metabolites

Microbial metabolites are compounds produced by intestinal microorganisms during their metabolic processes, which often play antiviral, antibacterial or anti‐inflammatory roles in fish. SCFAs are one of the common microbial metabolites, which can enhance the host’s antiviral ability by promoting the development and function of the immune system. Studies have shown that SCFAs in the intestinal tracts of fish can enhance the host’s immune response to viral infections. Butyrate protects Atlantic salmon against IPNV infection by reducing the replication of IPNV and modulating the immune response of pro‐ and anti‐inflammatory cytokines [120]. In addition to SCFAs, the gut microbiota can also inhibit viral infection by producing bile acids. The metabolite secondary bile acid (DCA) of *Parabacteroides distasonis* directly prevents the replication of SVCV and other Rhabdoviridae members in host fish [85]. The exopolysaccharides of *Cetobacterium somerae* inhibit SVCV infection through binding to the TLR2 receptor, thereby initiating the downstream type I IFN response [89]. Moreover, the production of diglyceride (DG) during lipid metabolism by *Bacillus* spp combats largemouth bass virus (LMBV) infection in teleost fish by activating key immune signaling pathways such as NF‐κB, thereby upregulating the expression of antiviral genes [95]. In conclusion, the role of microbial metabolites in fish viral diseases is multifaceted, involving immune regulation and direct immune signal regulation. These studies provide a new perspective for us to understand the complex role of microbial metabolites in fish viral diseases and offer potential targets for the development of new disease prevention and control strategies.

Although many studies suggest that microbiota interventions are crucial for preventing and managing viral diseases in fish, there are some limitations. Current research is often done in controlled labs with uniform fry, not reflecting the diverse and variable conditions of aquaculture. The absence of long‐term studies hinders the evaluation of sustained effects, potential side effects like dysbiosis, and contributions to antibiotic resistance. Additionally, introducing nonnative microorganisms may impact local ecosystems, and risks like horizontal gene transfer need thorough biosafety evaluations.

## 6. Conclusion and Future Prospects

The host, symbiotic bacteria, and viruses are engaged in a process of coevolution, resulting in a dynamic interplay among them. The impact of the gut microbiota on viral infections in fish represents a complex “double‐edged sword.” It can provide protective effects but may also exacerbate viral infection under certain conditions. The inhibitory effects of the gut microbiota on viruses are primarily manifested in its role in maintaining the stability of the fish’s intestinal immune system, which is achieved through various mechanisms, such as enhancing immune responses, competitively inhibiting the growth of pathogenic microorganisms, and strengthening intestinal barrier function, thereby resisting viral infection. Simultaneously, the gut microbiota may also act as a catalyst for viral infection. When the gut microbiota becomes imbalanced, it could decline host immune response, making fish more susceptible to viruses. Furthermore, preliminary research indicates a potential for direct interactions between certain harmful bacteria and viruses that could facilitate transmission. In addition, specific gut microbes could modulate the host’s immune response in a way that inadvertently creates a favorable environment for the virus, enhancing viral replication and spread. Therefore, in‐depth exploration of the interactions between viruses, hosts, and gut microbiota is warranted to distinguish correlation from causation. Revealing the specific pathways through which gut microbiota regulate immune responses will provide new targets for fish viral disease prevention and control strategies. Some viral infections are closely related to specific changes in the gut microbiota. By regulating the gut microbiota or intervening in the interactions between viruses and the microbiota, it could be possible to block viral infection pathways. Exploring the interactions between specific microorganisms in the gut and viruses can help identify strains with antiviral effects. Researching the impact of specific bacterial metabolites on viral infections will contribute to the development of antiviral therapies based on metabolic regulation. At the same time, research on gut microbiota and fish viral diseases faces numerous challenges. Although various techniques are currently used to study the gut microbiota and viral diseases, there are still some limitations. For example, existing technologies lack sufficient sensitivity when detecting certain low‐abundance microorganisms or viruses, making it difficult to fully understand the dynamic changes of microbial communities. Additionally, the complex interaction mechanisms between the gut microbiota and viruses have not been completely clarified, posing challenges for precise prevention and control. However, these challenges also present opportunities. With continuous technological innovation and development, new research methods and tools are emerging, providing possibilities for deeper investigations. High‐resolution single‐cell sequencing technologies, metatranscriptomics, and other advanced techniques can enable a more detailed study of the interactions between gut microbiota and viruses at the single‐cell level. At the same time, the interdisciplinary research trend offers new approaches to solving complex issues. By combining microbiology, immunology, genetics, bioinformatics, and other disciplines, we are likely to comprehensively reveal the relationship between gut microbiota and viral diseases, leading to the development of more effective prevention and control strategies. The gut microbiota is integral to the pathogenesis and regulation of viral diseases in fish. Although there is increasing interest in this area, current research predominantly remains correlative, with limited mechanistic insights. Future investigations should utilize germ‐free models and fecal microbiota transplantation to substantiate the causal roles of specific bacterial taxa. For instance, it is crucial to assess whether microbiota from resistant fish can confer protection. Comprehensive multi‐omics approaches are essential to elucidate the mechanisms by which microbial metabolites influence antiviral immunity. The development of probiotics must advance beyond traditional strains such as *Lactobacillus* and *Bacillus*, focusing instead on the identification of next‐generation probiotics with specific immune‐enhancing or antiviral properties. Synthetic biology offers the potential to engineer probiotics capable of continuously delivering antiviral peptides or immunomodulatory compounds. Considering the limitations of probiotics alone, the development of effective synbiotics is necessary to enhance colonization and functional activity. Large‐scale trials in commercial aquaculture are imperative to assess the efficacy of integrated strategies, such as the combination of probiotics/prebiotics with vaccines, in reducing mortality and viral load. Furthermore, since microbiota composition is influenced by species, age, diet, and environmental factors, future work should establish baseline “healthy microbiota” profiles for different aquaculture systems and develop tailored management strategies. In summary, advancing from correlation to mechanism, innovating in microbial intervention tools, and validating approaches in real‐world settings will be key to harnessing gut microbiota for sustainable viral disease control in aquaculture.

## Author Contributions

**Qiong Zhao:** conceptualization, investigation, visualization, writing – original draft, writing – review and editing. **Jianzhong Shao:** conceptualization, supervision. **Ye Chen:** conceptualization, funding acquisition, supervision, project administration, writing – review and editing, supervision. **Hangjun Zhang:** conceptualization, writing – review and editing, supervision.

## Funding

The study was supported by the China Postdoctoral Science Foundation (Grant 2025M772860), the Natural Science Foundation of China (Grant 32473191), and the Fundamental Research Funds for the Central Universities.

## Conflicts of Interest

The authors declare no conflicts of interest.

## Data Availability

The data that support the findings of this study are available upon request from the corresponding author. The data are not publicly available due to privacy or ethical restrictions.

## References

[1] Cao L. , Naylor R. , and Henriksson P. , et al.China’s aquaculture and the world’s wild fisheries, Science. (2015) 347, no. 6218, 133–135, 10.1126/science.1260149, 2-s2.0-84923326826. 25574011

[2] Weinstock G. M. , Genomic Approaches to Studying the Human Microbiota, Nature. (2012) 489, no. 7415, 250–256, 10.1038/nature11553, 2-s2.0-84866174523. 22972298 PMC3665339

[3] Meng K.-F. , Ding L.-G. , and Wu S. , et al.Interactions Between Commensal Microbiota and Mucosal Immunity in Teleost Fish During Viral Infection With SVCV, Frontiers in Immunology. (2021) 12, 10.3389/fimmu.2021.654758, 654758. 33897703 PMC8058427

[4] Huang Z. , Zhan M. , and Cheng G. , et al.IHNV Infection Induces Strong Mucosal Immunity and Changes of Microbiota in Trout Intestine, Viruses. (2022) 14, no. 8, 10.3390/v14081838, 1838. 36016461 PMC9415333

[5] Wang T. , Zhou N. , and Ding F. , et al.Xylanase Enhances Gut Microbiota-Derived Butyrate to Exert Immune-Protective Effects in a Histone Deacetylase-Dependent Manner, Microbiome. (2024) 12, no. 1, 10.1186/s40168-024-01934-6, 212. 39434145 PMC11492574

[6] López Nadal Aà , Ikeda-Ohtsubo W. , and Sipkema D. , et al.Feed, Microbiota, and Gut Immunity: Using the Zebrafish Model to Understand Fish Health, Frontiers in Immunology. (2020) 11, 10.3389/fimmu.2020.00114, 114. 32117265 PMC7014991

[7] Berger A. K. and Mainou B. A. , Interactions Between Enteric Bacteria and Eukaryotic Viruses Impact the Outcome of Infection, Viruses. (2018) 10, no. 1, 10.3390/v10010019, 2-s2.0-85042678033, 19. 29301335 PMC5795432

[8] Turner J. W.Jr., Cheng X. , Saferin N. , Yeo J.-Y. , Yang T. , and Joe B. , Gut Microbiota of Wild Fish as Reporters of Compromised Aquatic Environments Sleuthed Through Machine Learning, Physiological Genomics. (2022) 54, no. 5, 177–185, 10.1152/physiolgenomics.00002.2022. 35442774 PMC9126214

[9] Wong S. and Rawls J. F. , Intestinal Microbiota Composition in Fishes Is Influenced by Host Ecology and Environment, Molecular Ecology. (2012) 21, no. 13, 3100–3102, 10.1111/j.1365-294X.2012.05646.x, 2-s2.0-84862737884. 22916346 PMC4846280

[10] Sullam K. E. , Essinger S. D. , and Lozupone C. A. , et al.Environmental and Ecological Factors That Shape the Gut Bacterial Communities of Fish: A Meta-Analysis, Molecular Ecology. (2012) 21, no. 13, 3363–3378, 10.1111/j.1365-294X.2012.05552.x, 2-s2.0-84862770956. 22486918 PMC3882143

[11] Zhang J. , Liu Y. , and Shan S. , et al.Variation in the Gut Microbiota During the Early Developmental Stages of Common Carp (*Cyprinus carpio* L.) and its Correlation With Feed and Pond Water Microflora, BMC Veterinary Research. (2024) 20, no. 1, 10.1186/s12917-024-04321-3, 464. 39394135 PMC11468302

[12] Bledsoe J. W. , Peterson B. C. , Swanson K. S. , Small B. C. , and Rawls J. F. , Ontogenetic Characterization of the Intestinal Microbiota of Channel Catfish Through 16S rRNA Gene Sequencing Reveals Insights on Temporal Shifts and the Influence of Environmental Microbes, PLOS ONE. (2016) 11, no. 11, 10.1371/journal.pone.0166379, 2-s2.0-84995390625, e0166379. 27846300 PMC5113000

[13] Kashinskaya E. N. , Simonov E. P. , Kabilov M. R. , Izvekova G. I. , Andree K. B. , and Solovyev M. M. , Diet and Other Environmental Factors Shape the Bacterial Communities of Fish Gut in an Eutrophic Lake, Journal of Applied Microbiology. (2018) 125, no. 6, 1626–1641, 10.1111/jam.14064, 2-s2.0-85053892538. 30091826

[14] Sheng Y. , Ren H. , and Limbu S. M. , et al.The Presence or Absence of Intestinal Microbiota Affects Lipid Deposition and Related Genes Expression in Zebrafish (*Danio rerio*), Frontiers in Microbiology. (2018) 9, 10.3389/fmicb.2018.01124, 2-s2.0-85047668427, 1124. 29896183 PMC5987169

[15] Escalas A. , Troussellier M. , and Yuan T. , et al.Functional Diversity and Redundancy Across Fish Gut, Sediment and Water Bacterial Communities, Environmental Microbiology. (2017) 19, no. 8, 3268–3282, 10.1111/1462-2920.13822, 2-s2.0-85024376931. 28618142

[16] Pérez T. , Balcázar J. L. , and Ruiz-Zarzuela I. , et al.Host-Microbiota Interactions Within the Fish Intestinal Ecosystem, Mucosal Immunology. (2010) 3, no. 4, 355–360, 10.1038/mi.2010.12, 2-s2.0-77953787647. 20237466

[17] Xiao F. , Zhu W. , and Yu Y. , et al.Interactions and Stability of Gut Microbiota in Zebrafish Increase With Host Development, Microbiology Spectrum. (2022) 10, no. 2, 10.1128/spectrum.01696-21, e0169621. 35311546 PMC9045336

[18] Xin G.-Y. , Li W.-G. , Suman T. Y. , Jia P.-P. , Ma Y.-B. , and Pei D.-S. , Gut Bacteria *Vibrio sp*. and *Aeromonas sp*. Trigger the Expression Levels of Proinflammatory Cytokine: First Evidence From the Germ-Free Zebrafish, Fish & Shellfish Immunology. (2020) 106, 518–525, 10.1016/j.fsi.2020.08.018. 32810528

[19] Xie M. , Xie Y. , and Li Y. , et al.Stabilized Fermentation Product of *Cetobacterium somerae* Improves Gut and Liver Health and Antiviral Immunity of Zebrafish, Fish & Shellfish Immunology. (2022) 120, 56–66, 10.1016/j.fsi.2021.11.017. 34780975

[20] Pacheco I. , Díaz-Sánchez S. , and Contreras M. , et al.Probiotic Bacteria With High Alpha-Gal Content Protect Zebrafish Against Mycobacteriosis, Pharmaceuticals. (2021) 14, no. 7, 10.3390/ph14070635, 635. 34208966 PMC8308674

[21] Zhao Q. , Chang H. , and Zheng J. , et al.A Novel Trmt5-Deficient Zebrafish Model With Spontaneous Inflammatory Bowel Disease-Like Phenotype, Signal Transduction and Targeted Therapy. (2023) 8, no. 1, 10.1038/s41392-023-01318-6, 86. 36849517 PMC9971238

[22] Liu Y. , Cao Y. , and Zhang Y. , et al.Intestinal Flora and Immunity Response to Different Viscous Diets in Juvenile Largemouth Bass, *Micropterus salmoides* , Fish & Shellfish Immunology. (2022) 127, 1012–1023, 10.1016/j.fsi.2022.06.054. 35863540

[23] Xue M. , Fu M. , and Zhang M. , et al.Aflatoxin B1 Induced Oxidative Stress and Gut Microbiota Disorder to Increase the Infection of Cyprinid Herpesvirus 2 in Gibel Carp (*Carassius auratus* Gibelio), Antioxidants. (2023) 12, no. 2, 10.3390/antiox12020306, 306. 36829867 PMC9952714

[24] Zhang B. , Yang H. , Cai G. , Nie Q. , and Sun Y. , The Interactions Between the Host Immunity and Intestinal Microorganisms in Fish, Applied Microbiology and Biotechnology. (2024) 108, no. 1, 10.1007/s00253-023-12934-1, 30. 38170313 PMC13497495

[25] Stagaman K. , Burns A. R. , Guillemin K. , and Bohannan B. J. M. , The Role of Adaptive Immunity as an Ecological Filter on the Gut Microbiota in Zebrafish, The ISME Journal. (2017) 11, no. 7, 1630–1639, 10.1038/ismej.2017.28, 2-s2.0-85015663034. 28304369 PMC5520148

[26] Zeng R. , Pan W. , and Lin Y. , et al.Development of a Gene-Deleted Live Attenuated Candidate Vaccine Against Fish Virus (ISKNV) With Low Pathogenicity and High Protection, IScience. (2021) 24, no. 7, 10.1016/j.isci.2021.102750, 102750. 34278259 PMC8261673

[27] Fang Q. , Attoui H. , Biagini J. F. P. , Zhu Z. , de Micco P. , and de Lamballerie X. , Sequence of Genome Segments 1, 2, and 3 of the Grass Carp Reovirus (Genus Aquareovirus, Family Reoviridae), Biochemical and Biophysical Research Communications. (2000) 274, no. 3, 762–766, 10.1006/bbrc.2000.3215, 2-s2.0-0034637088. 10924351

[28] Ahne W. , Bjorklund H. V. , Essbauer S. , Fijan N. , Kurath G. , and Winton J. R. , Spring Viremia of Carp (SVC), Diseases of Aquatic Organisms. (2002) 52, no. 3, 261–272, 10.3354/dao052261, 2-s2.0-0037059147. 12553453

[29] Wen J. , Xu Y. , Su M. , Lu L. , and Wang H. , Susceptibility of Goldfish to Cyprinid Herpesvirus 2 (CyHV-2) SH01 Isolated From Cultured Crucian Carp, Viruses. (2021) 13, no. 9, 10.3390/v13091761. PMC8473056 34578342

[30] Dixon P. , Paley R. , Alegria-Moran R. , and Oidtmann B. , Epidemiological Characteristics of Infectious Hematopoietic Necrosis Virus (IHNV): A Review, Veterinary Research. (2016) 47, no. 1, 10.1186/s13567-016-0341-1, 2-s2.0-84976644593, 63. 27287024 PMC4902920

[31] Li C. , Liu J. , Zhang X. , Yu Y. , Huang X. , Wei J. , and Qin Q. , Red Grouper Nervous Necrosis Virus (RGNNV) Induces Autophagy to Promote Viral Replication, Fish & Shellfish Immunology. (2020) 98, 908–916, 10.1016/j.fsi.2019.11.053. 31770643

[32] López-Muñoz A. , Sepulcre M. P. , and García-Moreno D. , et al.Viral Nervous Necrosis Virus Persistently Replicates in the Central Nervous System of Asymptomatic Gilthead Seabream and Promotes a Transient Inflammatory Response Followed by the Infiltration of IgM+ B Lymphocytes, Developmental & Comparative Immunology. (2012) 37, no. 3-4, 429–437, 10.1016/j.dci.2012.02.007, 2-s2.0-84862132167. 22402274

[33] Dopazo C. P. , The Infectious Pancreatic Necrosis Virus (IPNV) and its Virulence Determinants: What is Known and What Should Be Known, Pathogens. (2020) 9, no. 2, 10.3390/pathogens9020094, 94. 32033004 PMC7168660

[34] Iida T. and Sano M. , Koi Herpesvirus Disease, Uirusu. (2005) 55, no. 1, 145–151, 10.2222/jsv.55.145, 2-s2.0-33644818223. 16308541

[35] Skall H. F. , Olesen N. J. , and Mellergaard S. , Viral Haemorrhagic Septicaemia Virus in Marine Fish and its Implications for Fish Farming – a Review, Journal of Fish Diseases. (2005) 28, no. 9, 509–529, 10.1111/j.1365-2761.2005.00654.x, 2-s2.0-33645863916. 16266325

[36] Way K. , Haenen O. , and Stone D. , et al.Emergence of Carp Edema Virus (CEV) and its Significance to European Common Carp and Koi *Cyprinus carpio* , Diseases of Aquatic Organisms. (2017) 126, no. 2, 155–166, 10.3354/dao03164, 2-s2.0-85031784720. 29044045

[37] Leiva-Rebollo R. , Labella A. M. , Gémez-Mata J. , Castro D. , and Borrego J. J. , Fish Iridoviridae: Infection, Vaccination and Immune Response, Veterinary Research. (2024) 55, no. 1, 10.1186/s13567-024-01347-1, 88. 39010235 PMC11247874

[38] Deperasińska I. , Schulz P. , and Siwicki A. K. , Salmonid Alphavirus (SAV), Journal of Veterinary Research. (2018) 62, no. 1, 1–6, 10.2478/jvetres-2018-0001, 2-s2.0-85048039934. 29978121 PMC5957455

[39] Rimstad E. and Markussen T. , Infectious Salmon Anaemia Virus—Molecular Biology and Pathogenesis of the Infection, Journal of Applied Microbiology. (2020) 129, no. 1, 85–97, 10.1111/jam.14567. 31885186

[40] Fusianto C. K. , Becker J. A. , and Subramaniam K. , et al.Genotypic Characterization of Infectious Spleen and Kidney Necrosis Virus (ISKNV) in Southeast Asian Aquaculture, Transboundary and Emerging Diseases. (2023) 2023, no. 1, 10.1155/2023/6643006, 6643006. 40303689 PMC12017167

[41] Rao Y. and Su J. , Insights Into the Antiviral Immunity Against Grass Carp (*Ctenopharyngodon idella*) Reovirus (GCRV) in Grass Carp, Journal of Immunology Research. (2015) 2015, no. 1, 10.1155/2015/670437, 2-s2.0-84924103963, 670437. 25759845 PMC4337036

[42] Ji J. , Liao Z. , and Rao Y. , et al.Thoroughly Remold the Localization and Signaling Pathway of TLR22, Frontiers in Immunology. (2020) 10, 10.3389/fimmu.2019.03003, 3003. 32010127 PMC6978911

[43] Shao Y. , Zhao J. , Ren G. , Lu T. , Chen X. , and Xu L. , Early or Simultaneous Infection With Infectious Pancreatic Necrosis Virus Inhibits Infectious Hematopoietic Necrosis Virus Replication and Induces a Stronger Antiviral Response During Co-Infection in Rainbow Trout (*Oncorhynchus mykiss*), Viruses. (2022) 14, no. 8, 10.3390/v14081732, 1732. 36016354 PMC9414607

[44] Vennerström P. , Maunula L. , Välimäki E. , and Virtala A. M. , Presence of Viral Haemorrhagic Septicaemia Virus (VHSV) in the Environment of Virus-Contaminated Fish Farms and Processing Plants, Diseases of Aquatic Organisms. (2020) 138, 145–154, 10.3354/dao03454. 32162613

[45] Machat R. , Pojezdal L. , Piackova V. , and Faldyna M. , Carp Edema Virus and Immune Response in Carp (*Cyprinus carpio*): Current Knowledge, Journal of Fish Diseases. (2021) 44, no. 4, 371–378, 10.1111/jfd.13335. 33460151

[46] Lin L. , Chen S. , and Russell D. S. , et al.Analysis of Stress Factors Associated With KHV Reactivation and Pathological Effects From KHV Reactivation, Virus Research. (2017) 240, 200–206, 10.1016/j.virusres.2017.08.010, 2-s2.0-85028733491. 28860099

[47] Turner J. K. , Sakulpolwat S. , and Sukdanon S. , et al.Tilapia Lake Virus (TiLV) Causes Severe Anaemia and Systemic Disease in Tilapia, Journal of Fish Diseases. (2023) 46, no. 6, 643–651, 10.1111/jfd.13775. 36848441

[48] Youngnoi N. , Yamkasem J. , and Khemthong M. , et al.Granulocyte Tropism and Lymphocyte Depletion Highlight the Immunopathogenesis of Tilapia Lake Virus Infection in Nile Tilapia, Fish & Shellfish Immunology. (2025) 163, 10.1016/j.fsi.2025.110410, 110410. 40368167

[49] Zhang L. , Chen W. Q. , Hu Y. W. , Wu X. M. , Nie P. , and Chang M. X. , TBK1-Like Transcript Negatively Regulates the Production of IFN and IFN-Stimulated Genes Through RLRs-MAVS-TBK1 Pathway, Fish & Shellfish Immunology. (2016) 54, 135–143, 10.1016/j.fsi.2016.04.002, 2-s2.0-84962883607. 27060200

[50] Hou G. , Lv Z. , and Liu W. , et al.An Aquatic Virus Exploits the IL6-STAT3-HSP90 Signaling Axis to Promote Viral Entry, PLOS Pathogens. (2023) 19, no. 4, 10.1371/journal.ppat.1011320, e1011320. 37099596 PMC10166480

[51] Reichert M. , Borzym E. , Matras M. , Maj-Paluch J. , Stachnik M. , and Palusinska M. , Down-Regulation of MHC Class I mRNA Expression in the Course of KHV Infection, Journal of Fish Diseases. (2016) 39, no. 10, 1253–1256, 10.1111/jfd.12451, 2-s2.0-84985996016. 26776370

[52] Chai W. , Qi L. , and Zhang Y. , et al.Evaluation of Cyprinid Herpesvirus 2 Latency and Reactivation in Carassius Gibel, Microorganisms. (2020) 8, no. 3, 10.3390/microorganisms8030445, 445. 32245260 PMC7143840

[53] Kim H. J. , Olesen N. J. , and Dale O. B. , et al.Pathogenicity of Two Lineages of Infectious Hematopoietic Necrosis Virus (IHNV) to Farmed Rainbow Trout (*Oncorhynchus mykiss*) in South Korea, Virus Research. (2023) 332, 10.1016/j.virusres.2023.199133, 199133. 37178795 PMC10345748

[54] Bergh Ø. , Boutrup T. S. , Johansen R. , Skall H. F. , Sandlund N. , and Olesen N. J. , Viral Haemorrhagic Septicemia Virus (VHSV) Isolated From Atlantic Herring, *Clupea harengus*, Causes Mortality in Bath Challenge on Juvenile Herring, Viruses. (2023) 15, no. 1, 10.3390/v15010152, 152. 36680192 PMC9866969

[55] Miyazaki T. , Kuzuya Y. , Yasumoto S. , Yasuda M. , and Kobayashi T. , Histopathological and Ultrastructural Features of Koi Herpesvirus (KHV)-Infected Carp *Cyprinus carpio*, and the Morphology and Morphogenesis of KHV, Diseases of Aquatic Organisms. (2008) 80, no. 1, 1–11, 10.3354/dao01929, 2-s2.0-47349126828. 18714678

[56] Huang X.-N. , Wang Z.-Y. , and Yao C.-L. , Characterization of Toll-Like Receptor 3 Gene in Large Yellow Croaker, Pseudosciaena Crocea, Fish & Shellfish Immunology. (2011) 31, no. 1, 98–106, 10.1016/j.fsi.2011.04.009, 2-s2.0-79957877120. 21549197

[57] Zhou Z.-X. , Zhang B.-C. , Sun L. , and Lang R. , Poly(I:C) Induces Antiviral Immune Responses in Japanese Flounder (*Paralichthys olivaceus*) That Require TLR3 and MDA5 and is Negatively Regulated by Myd88, PLoS ONE. (2014) 9, no. 11, 10.1371/journal.pone.0112918, 2-s2.0-84911905333, e112918. 25393122 PMC4231074

[58] Moss L. D. , Monette M. M. , and Jaso-Friedmann L. , et al.Identification of Phagocytic Cells, NK-Like Cytotoxic Cell Activity and the Production of Cellular Exudates in the Coelomic Cavity of Adult Zebrafish, Developmental and Comparative Immunology. (2009) 33, no. 10, 1077–1087, 10.1016/j.dci.2009.05.009, 2-s2.0-67650239145. 19477195

[59] Zapata A. , Diez B. , Cejalvo T. , Gutiérrez-de Frías C. , and Cortés A. , Ontogeny of the Immune System of Fish, Fish & Shellfish Immunology. (2006) 20, no. 2, 126–136, 10.1016/j.fsi.2004.09.005, 2-s2.0-22844440000. 15939627

[60] Kembou-Ringert J. E. , Steinhagen D. , Thompson K. D. , Daly J. M. , and Adamek M. , Immune Responses to Tilapia Lake Virus Infection: What We Know and What We Don’t Know, Frontiers in Immunology. (2023) 14, 10.3389/fimmu.2023.1240094, 1240094. 37622112 PMC10445761

[61] Wang Y.-Y. , Chen Y.-L. , and Ji J.-F. , et al.Negative Regulatory Role of the Spring Viremia of Carp Virus Matrix Protein in the Host Interferon Response by Targeting the MAVS/TRAF3 Signaling Axis, Journal of Virology. (2022) 96, no. 16, 10.1128/jvi.00791-22, e0079122. 35913215 PMC9400495

[62] Poynter S. , Lisser G. , Monjo A. , and DeWitte-Orr S. , Sensors of Infection: Viral Nucleic Acid PRRs in Fish, Biology. (2015) 4, no. 3, 460–493, 10.3390/biology4030460, 2-s2.0-84937682062. 26184332 PMC4588145

[63] Jensen S. and Thomsen A. R. , Sensing of RNA Viruses: A Review of Innate Immune Receptors Involved in Recognizing RNA Virus Invasion, Journal of Virology. (2012) 86, no. 6, 2900–2910, 10.1128/JVI.05738-11, 2-s2.0-84857969026. 22258243 PMC3302314

[64] Matsuo A. , Oshiumi H. , and Tsujita T. , et al.Teleost TLR22 Recognizes RNA Duplex to Induce IFN and Protect Cells From Birnaviruses, Journal of Immunology. (2008) 181, no. 5, 3474–3485, 10.4049/jimmunol.181.5.3474, 2-s2.0-51549108374. 18714020

[65] Byadgi O. , Puteri D. , Lee Y.-H. , Lee J.-W. , and Cheng T.-C. , Identification and Expression Analysis of Cobia (*Rachycentron canadum*) Toll-Like Receptor 9 Gene, Fish & Shellfish Immunology. (2014) 36, no. 2, 417–427, 10.1016/j.fsi.2013.12.017, 2-s2.0-84893206685. 24378677

[66] Priyathilaka T. T. , Elvitigala D. A. S. , and Whang I. , et al.Molecular Characterization and Transcriptional Analysis of Non-Mammalian Type Toll Like Receptor (TLR21) From Rock Bream (*Oplegnathus fasciatus*), Gene. (2014) 553, no. 2, 105–116, 10.1016/j.gene.2014.10.008, 2-s2.0-84908258880. 25300254

[67] Iliev D. B. , Sobhkhez M. , Fremmerlid K. , and Jørgensen J. B. , MyD88 Interacts With Interferon Regulatory Factor (IRF) 3 and IRF7 in Atlantic Salmon (*Salmo salar*): Transgenic SsMyD88 Modulates the IRF-Induced Type I Interferon Response and Accumulates in Aggresomes, Journal of Biological Chemistry. (2011) 286, no. 49, 42715–42724, 10.1074/jbc.M111.293969, 2-s2.0-82755176785. 21990356 PMC3234926

[68] Baoprasertkul P. , Peatman E. , Somridhivej B. , and Liu Z. , Toll-Like Receptor 3 and TICAM Genes in Catfish: Species-Specific Expression Profiles Following Infection With *Edwardsiella ictaluri* , Immunogenetics. (2006) 58, no. 10, 817–830, 10.1007/s00251-006-0144-z, 2-s2.0-33749656776. 16969679

[69] Lin K. , Ge H. , and Lin Q. , et al.Molecular Characterization and Functional Analysis of Toll-Like Receptor 3 Gene in Orange-Spotted Grouper (*Epinephelus coioides*), Gene. (2013) 527, no. 1, 174–182, 10.1016/j.gene.2013.06.014, 2-s2.0-84881237257. 23792060

[70] Samanta M. , Basu M. , Swain B. , Panda P. , and Jayasankar P. , Molecular Cloning and Characterization of Toll-Like Receptor 3, and Inductive Expression Analysis of Type I IFN, Mx and pro-Inflammatory Cytokines in the Indian Carp, Rohu (*Labeo rohita*), Molecular Biology Reports. (2013) 40, no. 1, 225–235, 10.1007/s11033-012-2053-6, 2-s2.0-84871330406. 23065215

[71] Chen S. N. , Zou P. F. , and Nie P. , Retinoic Acid-Inducible Gene I (RIG-I)-Like Receptors (RLRs) in Fish: Current Knowledge and Future Perspectives, Immunology. (2017) 151, no. 1, 16–25, 10.1111/imm.12714, 2-s2.0-85014285634. 28109007 PMC5382327

[72] Hiscott J. , Lin R. , Nakhaei P. , and Paz S. , MasterCARD: A Priceless Link to Innate Immunity, Trends in Molecular Medicine. (2006) 12, no. 2, 53–56, 10.1016/j.molmed.2005.12.003, 2-s2.0-32044457700. 16406812

[73] Loo Y.-M. and Gale M.Jr., Immune Signaling by RIG-I-Like Receptors, Immunity. (2011) 34, no. 5, 680–692, 10.1016/j.immuni.2011.05.003, 2-s2.0-79956314622. 21616437 PMC3177755

[74] Fang R. , Jiang Q. , and Zhou X. , et al.MAVS Activates TBK1 and IKKε Through TRAFs in NEMO Dependent and Independent Manner, PLOS Pathogens. (2017) 13, no. 11, 10.1371/journal.ppat.1006720, 2-s2.0-85036521808, e1006720. 29125880 PMC5699845

[75] Abe T. , Harashima A. , and Xia T. , et al.STING Recognition of Cytoplasmic DNA Instigates Cellular Defense, Molecular Cell. (2013) 50, no. 1, 5–15, 10.1016/j.molcel.2013.01.039, 2-s2.0-84876085954. 23478444 PMC3881179

[76] Cai X. , Chiu Y.-H. , and Chen Z. J. , The cGAS-cGAMP-STING Pathway of Cytosolic DNA Sensing and Signaling, Molecular Cell. (2014) 54, no. 2, 289–296, 10.1016/j.molcel.2014.03.040, 2-s2.0-84899131835. 24766893

[77] Cao J. , Xu H. , Yu Y. , and Xu Z. , Regulatory Roles of Cytokines in T and B Lymphocytes-Mediated Immunity in Teleost Fish, Developmental & Comparative Immunology. (2023) 144, 10.1016/j.dci.2022.104621, 104621. 36801469

[78] Zhu L.-Y. , Nie L. , Zhu G. , Xiang L.-X. , and Shao J.-Z. , Advances in Research of Fish Immune-Relevant Genes: A Comparative Overview of Innate and Adaptive Immunity in Teleosts, Developmental & Comparative Immunology. (2013) 39, no. 1-2, 39–62, 10.1016/j.dci.2012.04.001. 22504163

[79] Dixon B. and Stet R. J. M. , The Relationship Between Major Histocompatibility Receptors and Innate Immunity in Teleost Fish, Developmental & Comparative Immunology. (2001) 25, no. 8-9, 683–699, 10.1016/S0145-305X(01)00030-1, 2-s2.0-0034893866. 11602190

[80] Lee Y. K. and Mazmanian S. K. , Has the Microbiota Played a Critical Role in the Evolution of the Adaptive Immune System?, Science. (2010) 330, no. 6012, 1768–1773, 10.1126/science.1195568, 2-s2.0-78650647326. 21205662 PMC3159383

[81] Xu Z. , Takizawa F. , and Casadei E. , et al.Specialization of Mucosal Immunoglobulins in Pathogen Control and Microbiota Homeostasis Occurred Early in Vertebrate Evolution, Science Immunology. (2020) 5, no. 44, 10.1126/sciimmunol.aay3254. PMC7296778 32034088

[82] Dong S. , Ding L.-G. , and Cao J.-F. , et al.Viral-Infected Change of the Digestive Tract Microbiota Associated With Mucosal Immunity in Teleost Fish, Frontiers in Immunology. (2019) 10, 10.3389/fimmu.2019.02878, 2878. 31921142 PMC6930168

[83] Xiao F. , Liao L. , and Xu Q. , et al.Host-Microbiota Interactions and Responses to Grass Carp Reovirus Infection in Ctenopharyngodon Idellus, Environmental Microbiology. (2021) 23, no. 1, 431–447, 10.1111/1462-2920.15330. 33201573

[84] Reverter M. , Sarter S. , and Caruso D. , et al.Aquaculture at the Crossroads of Global Warming and Antimicrobial Resistance, Nature Communications. (2020) 11, no. 1, 10.1038/s41467-020-15735-6, 1870. PMC7170852 32312964

[85] Zhang Y. , Gao Y. , and Li C. , et al. *Parabacteroides distasonis* Regulates the Infectivity and Pathogenicity of SVCV at Different Water Temperatures, Microbiome. (2024) 12, no. 1, 10.1186/s40168-024-01799-9, 128. 39020382 PMC11253412

[86] Pereiro P. , Rey-Campos M. D. , Figueras A. , and Novoa B. , An Environmentally Relevant Concentration of Antibiotics Impairs the Immune System of Zebrafish (*Danio rerio*) and Increases Susceptibility to Virus Infection, Frontiers in Immunology. (2023) 13, 10.3389/fimmu.2022.1100092, 1100092. 36713462 PMC9878320

[87] Xie M. , Li Y. , and Olsen R. E. , et al.Dietary Supplementation of Exopolysaccharides From *Lactobacillus rhamnosus* GCC-3 Improved the Resistance of Zebrafish Against Spring Viremia of Carp Virus Infection, Frontiers in Immunology. (2022) 13, 10.3389/fimmu.2022.968348, 968348. 35990638 PMC9389081

[88] Zhang Y. , Zhao K. , and Liu Y. , et al.An Oral Probiotic Vaccine Loaded by *Lactobacillus casei* Effectively Increases Defense Against GCRV Infection in Grass Carp, Vaccine. (2025) 45, 10.1016/j.vaccine.2024.126660, 126660. 39729770

[89] Liang H. , Li M. , and Chen J. , et al.The Intestinal Microbiome and *Cetobacterium somerae* Inhibit Viral Infection Through TLR2-Type I IFN Signaling Axis in Zebrafish, Microbiome. (2024) 12, no. 1, 10.1186/s40168-024-01958-y. PMC11572407 39558430

[90] Lazado C. C. and Caipang C. M. A. , Mucosal Immunity and Probiotics in Fish, Fish & Shellfish Immunology. (2014) 39, no. 1, 78–89, 10.1016/j.fsi.2014.04.015, 2-s2.0-84901217975. 24795079

[91] Yin L. , Liu X. , and Yao Y. , et al.Gut Microbiota-Derived Butyrate Promotes Coronavirus TGEV Infection Through Impairing RIG-I-Triggered Local Type I Interferon Responses via Class I HDAC Inhibition, Journal of Virology. (2024) 98, no. 2, 10.1128/jvi.01377-23, e0137723. 38197629 PMC10878070

[92] Niu J. , Cui M. , and Yang X. , et al.Microbiota-Derived Acetate Enhances Host Antiviral Response via NLRP3, Nature Communications. (2023) 14, no. 1, 10.1038/s41467-023-36323-4, 642. PMC9901394 36746963

[93] Wang P. , Zhu S. , and Yang L. , et al.Nlrp6 Regulates Intestinal Antiviral Innate Immunity, Science. (2015) 350, no. 6262, 826–830, 10.1126/science.aab3145, 2-s2.0-84946922380. 26494172 PMC4927078

[94] She R. , Li T.-T. , and Luo D. , et al.Changes in the Intestinal Microbiota of Gibel Carp (*Carassius gibelio*) Associated With Cyprinid Herpesvirus 2 (CyHV-2) Infection, Current Microbiology. (2017) 74, no. 10, 1130–1136, 10.1007/s00284-017-1294-y, 2-s2.0-85026895893. 28748273

[95] Cheng G. , Kong W. , and Lin R. , et al.Multi-Omics Analysis Reveals That Bacillus spp. Enhance Mucosal Antiviral Immunity in Teleost Fish by Mediating Diglyceride Production Through Lipid Metabolism, Microbiome. (2025) 13, no. 1, 10.1186/s40168-025-02124-8, 123. 40380241 PMC12083065

[96] Syakuri H. , Adamek M. , and Brogden G. , et al.Intestinal Barrier of Carp (*Cyprinus carpio* L.) During a Cyprinid Herpesvirus 3-Infection: Molecular Identification and Regulation of the mRNA Expression of Claudin Encoding Genes, Fish & Shellfish Immunology. (2013) 34, no. 1, 305–314, 10.1016/j.fsi.2012.11.010, 2-s2.0-84871611577. 23194746

[97] Hai Q. , Wang J. , and Kang W. , et al.Metagenomic and Metabolomic Analysis of Changes in Intestinal Contents of Rainbow Trout (*Oncorhynchus mykiss*) Infected With Infectious Hematopoietic Necrosis Virus at Different Culture Water Temperatures, Frontiers in Microbiology. (2023) 14, 10.3389/fmicb.2023.1275649, 1275649. 37908544 PMC10614001

[98] Chen P. , Zhang M. , and Zhang Y. , et al.Cyprinid Herpesvirus 2 Infection Changes Microbiota and Metabolites in the Gibel Carp (*Carassius auratus* Gibelio) Midgut, Frontiers in Cellular and Infection Microbiology. (2023) 12, 10.3389/fcimb.2022.1017165, 1017165. 36817692 PMC9933507

[99] Piazzon M. C. , Naya-Català F. , Perera E. , Palenzuela O. , Sitjà-Bobadilla A. , and Pérez-Sánchez J. , Genetic Selection for Growth Drives Differences in Intestinal Microbiota Composition and Parasite Disease Resistance in Gilthead Sea Bream, Microbiome. (2020) 8, no. 1, 10.1186/s40168-020-00922-w, 168. 33228779 PMC7686744

[100] Wu S. , Wang G. , and Angert E. R. , et al.Composition, Diversity, and Origin of the Bacterial Community in Grass Carp Intestine, PLoS ONE. (2012) 7, no. 2, 10.1371/journal.pone.0030440, 2-s2.0-84857429885, e30440. 22363439 PMC3282688

[101] Ghosh A. K. , Functionality of Probiotics on the Resistance Capacity of Shrimp Against White Spot Syndrome Virus (WSSV), Fish & Shellfish Immunology. (2023) 140, 10.1016/j.fsi.2023.108942, 108942. 37451524

[102] Waiyamitra P. , Zoral M. A. , and Saengtienchai A. , et al.Probiotics Modulate Tilapia Resistance and Immune Response Against Tilapia Lake Virus Infection, Pathogens. (2020) 9, no. 11, 10.3390/pathogens9110919, 919. 33172079 PMC7694748

[103] Han S.-R. , Munang’andu H. M. , Yeo I.-K. , and Kim S.-H. , *Bacillus subtilis* Inhibits Viral Hemorrhagic Septicemia Virus Infection in Olive Flounder (*Paralichthys olivaceus*) Intestinal Epithelial Cells, Viruses. (2021) 13, no. 1, 10.3390/v13010028, 28. PMC7823535 33375689

[104] Liang X. , Liang J. , and Cao J. , et al.Oral Immunizations With *Bacillus subtilis* Spores Displaying VP19 Protein Provide Protection Against Singapore Grouper Iridovirus (SGIV) Infection in Grouper, Fish Shellfish Immunol. (2023) 138, 10.1016/j.fsi.2023.108860, 108860. 37257567

[105] Gao Y. , Huo X. , and Wang Z. , et al.Oral Administration of *Bacillus subtilis* Subunit Vaccine Significantly Enhances the Immune Protection of Grass Carp Against GCRV-II Infection, Viruses. (2022) 14, no. 1, 10.3390/v14010030, 30. PMC8779733 35062234

[106] Harikrishnan R. , Balasundaram C. , and Heo M.-S. , Effect of Probiotics Enriched Diet on *Paralichthys olivaceus* Infected With Lymphocystis Disease Virus (LCDV), Fish & Shellfish Immunology. (2010) 29, no. 5, 868–874, 10.1016/j.fsi.2010.07.031, 2-s2.0-77956271563. 20688170

[107] Liang H. , Xie Y. , and Li M. , et al.The Effect of Stabilized Culture of *Lactobacillus rhamnosus* GCC-3 on Gut and Liver Health, and Anti-Viral Immunity of Zebrafish, Fish & Shellfish Immunology. (2023) 141, 10.1016/j.fsi.2023.109074, 109074. 37714442

[108] Cui L.-C. , Guan X.-T. , Liu Z.-M. , Tian C.-Y. , and Xu Y.-G. , Recombinant Lactobacillus Expressing G Protein of Spring Viremia of Carp Virus (SVCV) Combined With ORF81 Protein of Koi Herpesvirus (KHV): A Promising Way to Induce Protective Immunity Against SVCV and KHV Infection in Cyprinid Fish via Oral Vaccination, Vaccine. (2015) 33, no. 27, 3092–3099, 10.1016/j.vaccine.2015.05.002, 2-s2.0-84937638115. 25981489

[109] Min L. , Li-Li Z. , Jun-Wei G. , Xin-Yuan Q. , Yi-Jing L. , and Di-Qiu L. , Immunogenicity of Lactobacillus-Expressing VP2 and VP3 of the Infectious Pancreatic Necrosis Virus (IPNV) in Rainbow Trout, Fish & Shellfish Immunology. (2012) 32, no. 1, 196–203, 10.1016/j.fsi.2011.11.015, 2-s2.0-84859236399. 22138084

[110] Galica A. N. , Galica R. , and Dumitrașcu D. L. , Diet, Fibers, and Probiotics for Irritable Bowel Syndrome, Journal of Medicine and Life. (2022) 15, no. 2, 174–179, 10.25122/jml-2022-0028. 35419092 PMC8999090

[111] Song S. K. , Beck B. R. , and Kim D. , et al.Prebiotics as Immunostimulants in Aquaculture: A Review, Fish & Shellfish Immunology. (2014) 40, no. 1, 40–48, 10.1016/j.fsi.2014.06.016, 2-s2.0-84903878896. 24973515

[112] Wang H. , Zhang X. , and Wang Z. , et al.Palmatine as a Potent Immunomodulator: Enhancing Resistance to *Micropterus salmoides* Rhabdovirus in Largemouth Bass Through Innate Immune Activation and Viral Suppression, Fish & Shellfish Immunology. (2024) 154, 10.1016/j.fsi.2024.109928, 109928. 39332654

[113] Qiao G. , Chen P. , and Sun Q. , et al.Poly-β-Hydroxybutyrate (PHB) in Bioflocs Alters Intestinal Microbial Community Structure, Immune-Related Gene Expression and Early Cyprinid Herpesvirus 2 Replication in Gibel Carp (*Carassius auratus* Gibelio), Fish & Shellfish Immunology. (2020) 97, 72–82, 10.1016/j.fsi.2019.12.045. 31846772

[114] Leal E. , Ordás M. C. , Soleto I. , Zarza C. , McGurk C. , and Tafalla C. , Functional Nutrition Modulates the Early Immune Response Against Viral Haemorrhagic Septicaemia Virus (VHSV) in Rainbow Trout, Fish & Shellfish Immunology. (2019) 94, 769–779, 10.1016/j.fsi.2019.09.070, 2-s2.0-85072832316. 31580935

[115] Krishnan R. , Jang Y.-S. , and Oh M.-J. , Beta Glucan Induced Immune Priming Protects Against Nervous Necrosis Virus Infection in Sevenband Grouper, Fish & Shellfish Immunology. (2022) 121, 163–171, 10.1016/j.fsi.2022.01.005. 35017048

[116] Librán-Pérez M. , Costa M. M. , Figueras A. , and Novoa B. , β-Glucan Administration Induces Metabolic Changes and Differential Survival Rates After Bacterial or Viral Infection in Turbot (*Scophthalmus maximus*), Fish & Shellfish Immunology. (2018) 82, 173–182, 10.1016/j.fsi.2018.08.005, 2-s2.0-85051672248. 30081180

[117] Liang H. , Li Y. , and Li M. , et al.The Effect and Underlying Mechanism of Yeast β-Glucan on Antiviral Resistance of Zebrafish Against Spring Viremia of Carp Virus Infection, Frontiers in Immunology. (2022) 13, 10.3389/fimmu.2022.1031962, 1031962. 36405758 PMC9669391

[118] Medina-Gali R. M. , del Mar Ortega-Villaizan M. , Mercado L. , Novoa B. , Coll J. , and Perez L. , Beta-Glucan Enhances the Response to SVCV Infection in Zebrafish, Developmental & Comparative Immunology. (2018) 84, 307–314, 10.1016/j.dci.2018.02.019, 2-s2.0-85043465266. 29524446

[119] Djordjevic B. , Škugor S. , Jørgensen S. M. , Øverland M. , Mydland L. T. , and Krasnov A. , Modulation of Splenic Immune Responses to Bacterial Lipopolysaccharide in Rainbow Trout (*Oncorhynchus mykiss*) Fed Lentinan, a Beta-Glucan From Mushroom *Lentinula edodes* , Fish & Shellfish Immunology. (2009) 26, no. 2, 201–209, 10.1016/j.fsi.2008.10.012, 2-s2.0-60949084768. 19010422

[120] Vargas R. A. , Soto-Aguilera S. , and Parra M. , et al.Analysis of Microbiota-Host Communication Mediated by Butyrate in Atlantic Salmon, Computational and Structural Biotechnology Journal. (2023) 21, 2558–2578, 10.1016/j.csbj.2023.03.050. 37122632 PMC10130356

